# P04.69. Investing in integrative medicine for mental health and wellbeing: making the economic case

**DOI:** 10.1186/1472-6882-12-S1-P339

**Published:** 2012-06-12

**Authors:** D McDaid, A Park

**Affiliations:** 1 London School of Economics and Political Science, London, United Kingdom

## Purpose

One in four individuals can expect to experience a mental health problem during their lifetimes. This has a significant impact on the health system and wider economy. Annual costs of depression in the EU were more than €136.3 billion in 2007. These costs are likely to increase, with depression predicted to become the leading cause of morbidity in high income countries by 2030. Policy makers thus want increasing information on the costs and benefits of investing in mental health promotion.

## Methods

A rapid review of literature was undertaken to identify estimates of costs/effects of selected integrative approaches: mindfulness, meditation, Tai Chi, Qi-Gong and yoga. Decision-analytic modelling techniques were used to synthesise data on the costs/effectiveness of these integrative medicine approaches for the prevention and treatment of stress, anxiety and depressive disorders, as well as any benefits of improved mental wellbeing. Cost effectiveness data and net returns on investment over short (1 year); mid (5 year); and long (10 year) timeframes for improved mental health were estimated. This data were then compared with that calculated by the authors in a previous study undertaken for the Department of Health in England on a range of conventional interventions and other actions to promote mental health and wellbeing.

## Results

If integrative medicine can achieve modest improvements in depressive symptoms, economic modelling indicates positive returns on investment greater than 2:1 in the long run. This is comparable to some psychological therapies now rolled out. Much benefit is realised outside the healthcare sector, and is due to greater participation in work and other everyday activities.

## Conclusion

There is an economic case for greater consideration of the potential of mindfulness, meditation, Tai Chi, Qi-Gong and yoga as alternative or adjunct options both for the prevention and early intervention to treat mild and moderate stress, depression and anxiety disorders.

